# Exploring Cortical Thickness Alteration in Parkinson Disease Patients with Freezing of Gaits

**DOI:** 10.1155/2020/8874119

**Published:** 2020-11-27

**Authors:** E. Li, Xiuhang Ruan, Yuting Li, Guoqin Zhang, Mengyan Li, Xinhua Wei

**Affiliations:** ^1^Department of Radiology, Guangzhou First People's Hospital, Guangzhou Medical University, Guangzhou, Guangdong 510180, China; ^2^Department of Radiology, Guangzhou First People's Hospital, School of Medicine, South China University of Technology, Guangzhou, Guangdong 510180, China; ^3^Department of Neurology, Guangzhou First People's Hospital, School of Medicine, South China University of Technology, Guangzhou, Guangdong 510180, China

## Abstract

*Background:* Freezing of gait (FoG) is a disabling gait disorder that commonly occurs in advanced stages of Parkinson's disease (PD). The neuroanatomical mechanisms underlying FoG in PD are still unclear. The present study aims to explore alterations of structural gray matter (GM) in PD patients with FoG. *Method:* Twenty-four PD patients with FoG (FoG+), 37 PD patients without FoG (FoG-) and 24 healthy controls (HC) were included. All subjects underwent a standardized MRI protocol. The cortical thickness (CTh), segmentation volume without ventricles (BrainSegVolNotVent) and estimated total intracranial volume (eTIV) were analysed using the FreeSurfer pipeline. *Results:* CTh differences were found in the right middle temporal gyrus (rMTG) generally. Compared to that in HCs, the CTh of the rMTG in both the FoG+ and FoG- groups was smaller, while no significant difference between the FoG+ and FoG- groups. Correlation analyses demonstrated a negative correlation between the CTh of the rMTG and the UPDRS part II score in PD subjects, and a borderline significant correlation between the score of Freezing of Gait Questionnaire (FoGQ) and rMTG CTh. Additionally, receiver operating characteristic curve (ROC) analysis revealed a cut-off point of CTh =3.08 mm in the rMTG that could be used to differentiate PD patients and HCs (AUC =0.79, P <0.01). There were no differences in the BrainSegVolNotVent or eTIV among the 3 groups. *Conclusions:* Our findings currently suggest no significant difference between FoG+ and FoG- patients in terms of structural gray matter changes. However, decreased CTh in the rMTG related to semantic control may be used as a biomarker to differentiate PD patients and HCs.

## 1. Introduction

Parkinson's disease (PD) remains as a challenging health problem, especially among elderly people, worldwide [1]. Approximately 17/1000 people above the age of 65 years old suffer from Parkinson's disease [2]. Freezing of gait (FoG), defined as a brief, episodic restriction or marked reduction of forward progression of the feet despite the intention to walk [3], is one of the most notable characteristics of advanced Parkinson's disease. It may cause patients decreased mobility, reduction in quality of life, and even bring about accidents due to falling. However, the mechanism behind FoG has yet to be fully discovered. A more concrete explanation of mechanisms behind FoG will not only guide early prevention and cure of this phenomenon but also help to understand the physiology of normal locomotion generation, control and management in humans.

Currently, Parkinson's disease is widely understood as a neurodegenerative disease [4]. There is evidence that degeneration of cholinergic neurons in PD patients may contribute to the onset of FoG [5]. Fortunately, magnetic resonance imaging (MRI) has provided us with various methods to investigate neural degeneration in vivo. Recently, MRI-based quantitative neuroanatomical approaches have been used to assess various disorders. In particular, cortical thickness (CTh), measured as the width of the cortical gray matter layers that cover the surface of the brain, has shown conspicuous advantages compared to other parameters, and has been considered as a possible biomarker for quite a few neurological disorders [6–8]. It is able to reach sub-voxel level precision, enabling more accurate measurement in deep sulci [9]. Moreover, changes in cortical thickness may occur before neuronal death, supporting the indication capability of CTh in diseases' preclinical stages [10]. There are already a handful of studies using cortex-based method to explore specific clinical-relevant situations in PD. Previous studies have found that CTh reduction in frontal, temporal, parietal and occipital areas occurs in mild cognitive impairment (MCI) in early PD, while disease stage is negatively correlated with CTh in multiple brain regions [11]. Therefore, exploring possible role of CTh as a biomarker in PD with FoG and establishing disease model therewith may facilitate early detection of the situation at quantitative level, enabling early intervention at precise brain circuits affected, thus improving quality of life, preventing accidents related to motor disabilities.

To the best of our knowledge, at the time of this study, only 2 studies had explored the cortical pattern of freezing of gait with cortex-based methods. One study discovered CTh reduction in the left supplementary motor area, middle/anterior cingulate cortex, temporal pole and right frontal operculum of FoG positive PD patients in contrast to FoG negative ones. However, the study did not include healthy control participants [12]. The other study has found cortex degeneration in superior frontal gyrus, paracentral lobule, posterior cingulate cortex, precuneus and pericalcarine cortex, as well as right dorsolateral prefrontal cortex in comparison with HC, but no significant difference was found between FoG positive and FoG negative subjects [13]. The discrepancy of their findings may possibly be related to differences of details in cortex-based analysis processes, as well as variance in disease severity between the two populations of patients. This inevitably resulted in an ambiguous understanding of the neuroanatomical features behind FoG. Meanwhile, both of the two studies focused on Caucasian subjects, which implies that this may be the first study to investigate Asian population about PD FoG so far.

In this study, we planned to use the structural gray matter method to explore possible neural degeneration in both hemispheres in PD patients with FoG and tried to analyse the relationship of cortical thickness and disease severity. Additionally, we tried to identify whether the alteration of CTh in brain regions present is specific to FoG positive patients with receiver operating characteristic curve (ROC) analysis.

## 2. Method

### 2.1. Participants

This study was approved by the Ethics Committee of Guangzhou First People's Hospital. All participants provided written informed consent before examination. Sixty-one patients with Parkinson's disease in Guangzhou First People's Hospital were selected from 2017.3 to 2018.11. Inclusion criteria were as the following: (1) Diagnosed with PD according to the United Kingdom PD Society Brain Bank criteria; and (2) Right-handed as defined by the Edinburgh Handedness Inventory. Patients were excluded when the following situations were present: (1) Stage 5 on the Hoehn and Yahr scale (H-Y); (2) Secondary PD syndrome due to other reasons; (3) Inability to cooperate for the MRI scanning procedures due to severe motion disorder; (4) History of other neural system diseases, such as stroke or brain trauma; (5) History of severe disease of the motor system, such as osteoarthropathy, osteomyopathy or arthroplasty; (6) History of psychiatric disorders; (7) Presence of implanted objects in the head such as false teeth, which may cause artefacts in the image.

Patients were recognized as FoG+ if they scored >0 on questions 3-6 of the Freezing of Gait Questionnaire (FoGQ), along with clinical diagnosis by a professional neurologist (M.L, with >20 years experiences). Twenty-four PD patients were defined as freezing of gait positive (FoG+), and 37 PD patients were freezing of gait negative (FoG-). Disease stages were then assessed in two groups of PD subjects by the H-Y scale. General daily performance and drug complications were evaluated with Unified Parkinson's Disease Rating Scale (UPDRS) part I to IV, and cognitive status with Mini-Mental State Examination (MMSE). The presence and severity of FOG was evaluated by FOGQ.

The pharmacological treatment of both FoG+ and FoG- patients was recorded in the form of daily Levodopa equivalent dose (LED). The dose of dopamine replacement medication was converted by the formula in previous papers.

Twenty-four healthy controls (HCs) were also recruited in our study using the following inclusion criteria: (1) no severe clinical disease, (2) normal state of mind and self-management ability, (3) right-handed as defined by the Edinburgh Handedness Inventory. The exclusion criteria were as follows: (1) neuropsychiatric disorder, (2) recent intake of neuropsychiatric medicine in the previous 1 week before MRI examination, (3) self-reported cognitive impairment or MMSE<24, and (4) brain lesion >1 cm or moderate or greater white matter hyperintensity on MR images. The cognitive status of HCs was also assessed with MMSE.

### 2.2. Image Data Acquisition

Participants underwent an MRI scans using a 3.0 T MRI scanner (Siemens Verio, Erlangen, Germany) with a magnetization-prepared rapid acquisition gradient-echo (MPRAGE) sequence. The scanning protocol was as follows: repetition time (TR) =1900 ms, echo time (TE) =2.19 ms, flip angle =9°, field of view (FOV) = 250 × 250 mm, slice thickness of 1.0 mm, and 256 × 256 acquisition matrix. The time for a single scan was 6 minutes and 1 second. All image data acquired was visually inspected by a professional radiologist (X.R) to ensure quality. Image would only be used with no visible motion artifact as well as substantial contrast between grey matter and white matter. All PD subjects were scan under “ON” state.

### 2.3. Gray Matter Analysis

#### 2.3.1. Cortical Thickness Analysis

The analysis of cortical thickness was carried out with the FreeSurfer toolkit (http://surfer.nmr.mgh.harvard.edu). Firstly, the intensity of T1-weighted images was normalized. Then, a skull-strip algorithm removed the skull and all non-brain tissues (scalp, skins and eyeball) from the image, and a mask of gross brain tissue was created. The brain tissue of each subject was then segmented into different regions. Next, white matter and gray matter surfaces were tessellated, transforming 3D voxels of the brain into 2D curvatures. Boundaries of white matter and gray matter (pial surface) were then identified. Cortical thickness was defined as the shortest distance between the two boundaries above each vertex. The image data for the whole brain were sequentially inflated into a sphere and registered on the MNI template. After that, a smoothing process was performed with a 10-mm full-width at half maximum Gaussian kernel. Finally, image data for the cortex were mapped back to each individual according to sulci and gyri. All of the processes above were executed automatically in the FreeSurfer “recon-all” pipeline. The results after processing were visually inspected in case error and bias existed.

#### 2.3.2. Cortical Volume Analysis

The brain segmentation volume without ventricles (BrainSegVolNotVent) value and estimated total intracranial volume (eTIV) of each subject were also calculated by the pipeline.

### 2.4. Statistical Analysis

Comparison of age and the MMSE score were conducted with ANOVA and sex composition were compared using Chi-square test between the 3 groups. The difference in Disease duration, LED, H-Y score, UPDRS part I score, UPDRS part IV score and the FoGQ scores between FoG+ and FoG- groups of PD patients were analysed with Mann–Whitney *U* tests, and the UPDRS score of part II and III with t-tests.

A general linear model (GLM) analysis was applied to identify difference in CTh at vertex-wise scale between groups on both hemispheres, considering age, disease duration, eTIV and LED (in PD groups) as covariates. The main effect of CTh difference was corrected using false discovery rate (FDR) correction at q =0.05. The cortical thickness value of regions with significant differences was then extracted for each participant. We used t-tests to accomplish comparisons of CTh between groups in FoG+ Vs HC, FoG- Vs HC and FoG+ Vs FoG-, and one-way ANOVA for the contrast of BrainSegVolNotVent and eTIV. Statistical significance was defined as P <0.01.

Next, correlation analyses were performed between CTh and clinical evaluation data for the FoG+ and FoG- groups, respectively, as well as all the PD subjects as a whole, with age and disease duration as covariates.

We also performed ROC analyses to evaluate the classification value of CTh to determine whether it could differentiate PD (FoG+ and FoG-) patients from HCs or the FoG+ group from the FoG- group. The sensitivity, specificity, and optimal cut-off points were calculated. The standard of statistical significance were set as P <0.05 for the statistical analyses above.

## 3. Results

### 3.1. Demographic Information

The demographic and clinical assessments data for the 3 groups of participants are shown in Table 1. There was no significant difference among the 3 groups for age, sex, or MMSE score. Regarding the two groups of PD patients, FoG+ patients had a longer average disease duration, larger medication dose measured by LED than the FoG- group, as well as higher UPDRS scores on parts II and IV. Patients with FoG also had distinctly higher scores on the FoG questionnaire than those without.

### 3.2. Cortical Thickness and Volume-Based Results

**Figure 1:**
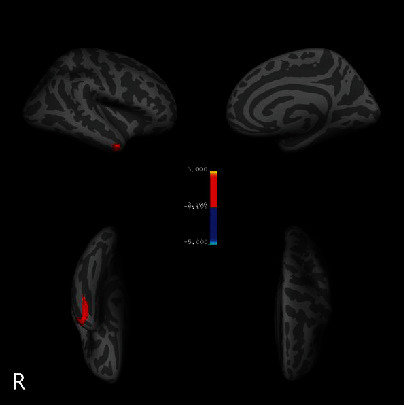
Significant reduction of cortical thickness was discovered in middle temporal gyrus (MTG) of right hemisphere at global level.

**Figure 2:**
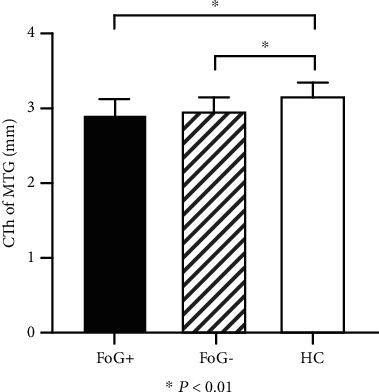
Comparison of CTh of right middle temporal gyrus (MTG) between groups. CTh was lower in PD FoG+ group and FoG- group compared with HC, but no significant difference between two groups of PD subjects.

Overall, we discovered cortical thickness reduction in the middle temporal gyrus of the right hemisphere (rMTG), which is located at MNI coordinates (50.6, 2.5, 33.0) (Figure 1). As the result of the post hoc test for between-group comparisons, the CTh of the rMTG was thinner in the FoG+ group (P <0.01) and FoG- group (P <0.01) than in the HC group (Figure 2). Meanwhile, we found that the CTh of the rMTG in FoG+ patients was to be thinner than that in FoG- patients, but the result was not statistically significant (P =0.42). There was no statistical difference in BrainSegVolNotVent (P =0.89) or eTIV (P =0.70) among the 3 groups.

### 3.3. ROC Analysis

**Figure 3:**
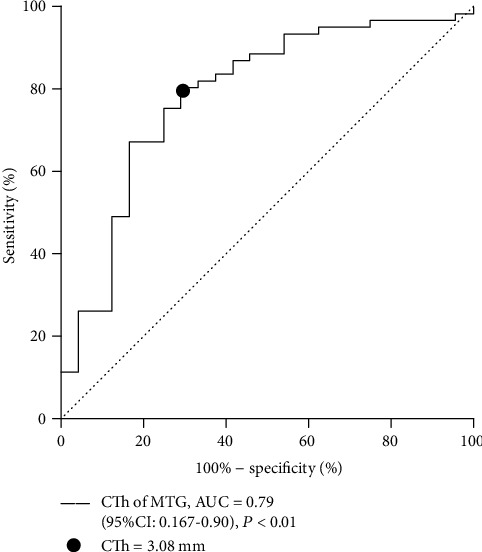
The ROC analysis showing the value of the CTh of right middle temporal gyrus (MTG) in differentiating PD patients and healthy controls. The black dot indicates the cut-off point when sensitivity+specificity-1 reach the maximum.

As a result, the CTh of the rMTG could not effectively separate the FoG+ group from the FoG- group at statistically significant level (P =0.16). However, when taking the whole PD patients sample (FoG+ and FoG- groups) into consideration, we found that the CTh of the rMTG was able to differentiate patients with PD from healthy controls (AUC =0.79, P <0.01) and that the best cut-off point was reached when CTh =3.08 mm, with a sensitivity of 80%, 95% CI: (0.75, 0.94) and specificity of 71%, 95% CI: (0.39, 0.76) (Figure 3).

### 3.4. Correlation between Clinical Stages

**Figure 4:**
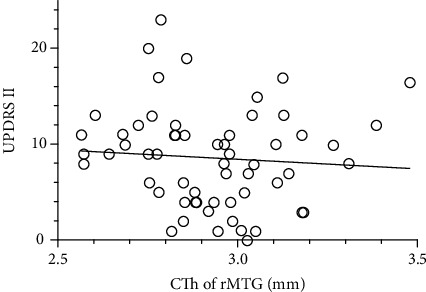
CTh of right middle temporal gyrus (MTG) showing negative correlation with UPDRS part II score in PD patients after correcting the influence of age and disease duration (P <0.05).

After correcting for the influence of age and disease duration, we discovered a negative correlation between the UPDRS part II score and the CTh of the rMTG (P <0.05) (Figure 4). Meanwhile, a borderline significance was found in the correlation of rMTG CTh and FOGQ (R^2^ = 0.433; P =0.072). No significant correlation was established between the CTh of the rMTG and other clinical assessments of disease stages (Table 2).

## 4. Discussion

In this study, we used a surface-based method to explore whole-brain cortical thickness in FoG+ and FoG- PD patients and healthy controls. As a result, a reduction in CTh was present in the rMTG among all PD patients compared with HC, and its value was negatively correlated with the UPDRS part II score. Additionally, ROC analysis showed that it may effectively separate patients with PD from HCs.

The possible relationship between middle temporal gyrus (MTG) and Parkinson's disease has been described in many previous studies. Gao et al. discovered CTh reduction in the middle temporal cortex along with the fusiform gyrus, insula of the left hemisphere and fusiform gyrus, isthmus cingulate cortex, inferior temporal gyrus and posterior cingulate cortex of the right hemisphere in PD patients, while the CTh of the left temporal pole was correlated with UPDRS-III scores. Yang et al. [14] reported that significant increased activity was observed in the right middle temporal lobe in early PD. The role of the MTG in brain function has not yet been fully discovered, but a few functional and anatomical studies have provided important hints about its responsibility. Tractography-based analysis indicated that the middle temporal gyrus can be divided into 4 parts: 1) the anterior middle temporal gyrus (aMTG), 2) the middle part of the middle temporal gyrus (mMTG), 3) the posterior middle temporal gyrus (pMTG) and 4) the sulcus of the middle temporal gyrus (sMTG) [15]. Respectively, the aMTG is a crucial part of the DMN while participates in language comprehension. The mMTG acts as an important hub in the formation of semantic memory and retrieval of social knowledge. The pMTG takes part in language processing, being indispensable when people read or repeat tasks, while the sMTG helps to perceive social attention cues from others, including body posture, head position or eye gaze direction. This strongly supports results from quite a few studies indicating that cognitive impairment in PD significantly correlates with degeneration or deactivation of the MTG [16–18].

A neural tracing experiment using an injected tracer in the anterior intraparietal area (AIP) of macaques showed that the MTG has additional connectivity with the AIP, indicating that it also plays an important role in object recognition by connecting the ventral visual stream with the hand motor system [19]. In our study, the UPDRS part II score was higher in PD patients with FoG than in those without FoG, while negatively correlated with the CTh of the rMTG. This part of score evaluated disease stage by evidence including the use of tableware, dressing, and daily hygiene, which obviously involve object recognition and operation, as well as retrieval of language messages. Therefore, our findings help to understand the deteriorated ability to perform daily activities in FoG+ patients from a different perspective.

Although the mechanism behind FoG has yet to be fully understood, researchers have already made a number of plausible hypotheses about its origin. One of the most widely accepted models attributes FoG to the disturbance of the motor system in charge of balance and locomotion [5]. But, in fact, the motor network is very intricate, with a wide range of connections to various functional regions of the brain. Afferent feedback from sensory systems, along with attentional and emotional network status, could exert a significant influence on the symptoms of FoG [20]. Meanwhile, in clinical practice, symptoms of FoG+ patients can always be alleviated by giving auditory instructions and emotional cueing about proper steps [21]. Pelosin et.al successfully improve motor performance of PD FoG patients with action observation training (AOT) [22], indicating that retrieving semantic information may actually exert compensatory effect on the motor system. A previous fMRI study found that the left middle temporal and middle occipital gyri and the inferior, middle and superior frontal regions were activated almost exclusively in motor ideation, giving us additional evidence that degeneration of the MTG cortex may contribute to motor dysfunction in PD. However, out of our expectation, the final result did not provide significant evidence for aberrant cortical alteration at MTG specifically in FoG+ patients. We speculated several possible causes which might lead to such outcomes: 1. The sample size of our study is rather small, with merely 24 FoG+ participants and 37 FoG- ones. This would inevitably limit the statistical power in the analytical processes. 2. Studies pointed out that freezing of gait could be further categorized into 3 subtypes according to specific patterns of leg motion: (1) shuffling with small steps, (2) trembling in place, (3) complete akinesia [23]. And there is already evidence about gray matter structural alteration between motor subtypes of Parkinson's disease (not freezing of gaits) [24]. However, our study only considered the situation as “positive” and “negative” status of FoG, possibly covering the existing differences between the 3 subtypes. 3. Although the effect of medication dose (measured by LED) was controlled in the statistical processes, application of levodopa is still likely to shape brain networks in structural and functional levels under long-term effect [25, 26]. In this study, average LED of FoG positive group was larger than those negative ones, which might implicate medication effect on cortical structural changes. However, due to longer disease duration and more severe symptom, FoG positive PD patients are always to be treated with higher accumulative dosage of medication, which constitute the difficulty to find dosage-matched participants. 4. Additionally, differences in detail of image data processing and analyzing, such as standard of quality control and proper parameters setting according to data characteristic, may also contribute to the discrepancy. Therefore, future research will be necessary to advance our exploration with adequate sample size under similar medication dosage among more specific FoG subtypes, as well as more controlled processes in data analysis.

In quantitative morphometric brain studies, the results can always be influenced by head size variation between participants. In one volume-based study concerning idiopathic PD, differences in the volume of brain regions were altered significantly before and after controlling for total intracranial volume (TICV) [27]. In FreeSurfer, total intracranial volume is represented by an automated calculated parameter deduced from the Atlas Scaling Factor (ASF), aka estimatedEstimated total intracranial volume (eTIV) [28]. The results showed no difference presented in BrainSegVolNotVent and eTIV between groups in our study, indicating that data from different groups were comparable without correction of whole brain volume.

Our study still has many limitations. Firstly, as mentioned before, our sample size is rather small, which is one of the main drawbacks of this study. This is partially due to our requirement for matched participants and the relatively short duration of the study. Secondly, using CTh of rMTG as a biomarker for PD should be resample by replication groups. Due to the small sample size of our study, we were unable to accomplish this procedure. Thirdly, all of our participants were right-handed. This may cause bias in laterality in our results. Therefore, our results should be resampled in future research with a larger population and participants with both handedness. Moreover, researchers may focus on exploring the intrinsic relationship between the semantic system and freezing of gait from different perspectives, such as fMRI and DTI, and try to understand how the visual-motor system and semantic system are interwoven.

In summary, we explored possible neural degeneration in PD patients with FoG using the cortical thickness method. We discovered that the CTh of the rMTG is thinner in Parkinson's disease patients than in HC, while it is negative correlated with the UPDRS part II score, and correlation was borderline-significant between FOGQ and rMTG CTh. However, the difference of CTh was not significant between FoG+ and FoG- PD subjects in our study. Additionally, the CTh of the rMTG could effectively differentiate PD subjects from HC, but not FoG positive patients from those negative ones. We speculate that degeneration of brain regions in charge of the semantic control system may participate in the presence of motor disabilities in PD. Further researches may try to validate our findings with more substantial sample size, as well as to determine the relationship between semantic control and the visual-motor system following cues from our study.

## Figures and Tables

**Table 1:** Demographics and Clinical evaluations of 3 groups of participants.

Variables	FoG+(n =24)	FoG-(n =37)	HC(n =24)	P
Gender (males/females)	13/11	19/18	9/15	0.456
Age(years)	65.5 ± 6.1	64.1±8.2	62.5±3.8	0.298
MMSE	25.3 ± 4.1	25.7±4.3	27.6±2.1	0.105
Disease duration(years)	6 ± 5.3	3±3.2		0.003∗
H-Y	2.5 ± 0.5	2.07±0.5		0.012
LED (mg/d)	592 ± 137	310±74		<0.001∗
UPDRS I	2.0 ± 2.3	1.0±1.5		0.060
UPDRS II	11.4 ± 7.0	7.4±4.2		0.007∗
UPDRS III Total	29.3 ± 17.3	28.0±13.2		0.741
UPDRS IV	2.9 ± 3.0	0.73±1.4		0.001∗
FOG Q	8.4±4.9	1.4±1.3		<0.001∗

Value of each category represent the means (±1 SD). ∗:P <0.05.

**Table 2:** Linear regression results between CTh of rMTG and clinical evaluations.

Variables	*R* ^2^	*P*
MMSE	0.012	0.800
H-Y	0.608	0.580
UPDRS I	0.230	0.144
UPDRS II	0.575	0.018∗
UPDRS III Total	0.296	0.830
UPDRS IV	0.621	0.311
FOG Q	0.433	0.072

R2 represents the coefficient of determination in the correlation analysis of each variable.∗:P <0.05.

## Data Availability

Our data may be available upon reasonable request. Please contact weixinhua@aliyun.com for details.
